# Dog Aging: A Comprehensive Review of Molecular, Cellular, and Physiological Processes

**DOI:** 10.3390/cells13242101

**Published:** 2024-12-18

**Authors:** Gabriella Guelfi, Camilla Capaccia, Martina Tedeschi, Antonello Bufalari, Leonardo Leonardi, Beniamino Cenci-Goga, Margherita Maranesi

**Affiliations:** Department of Veterinary Medicine, University of Perugia, Via San Costanzo 4, 06126 Perugia, Italy; camilla.capaccia@dottorandi.unipg.it (C.C.); martina.tedeschi@studenti.unipg.it (M.T.); leonardo.leonardi@unipg.it (L.L.); beniamino.cencigoga@unipg.it (B.C.-G.); margherita.maranesi@unipg.it (M.M.)

**Keywords:** physiological decline, aging dog, aging hallmarks, epigenetic changes

## Abstract

The aging process is a multifactorial biological phenomenon starting at birth and persisting throughout life, characterized by a decline in physiological functions and adaptability. This decline results in the diminished capacity of aging organisms to respond to environmental changes and stressors, leading to reduced efficiency in metabolic, immune, and hormonal functions. As behavioral flexibility wanes, older individuals face longer recovery times and increased vulnerability to diseases. While early research proposed nine core hallmarks of mammalian aging, recent studies have expanded this framework to twelve key characteristics: epigenetic changes, genomic instability, telomere shortening, loss of proteostasis, altered metabolism, mitochondrial dysfunction, cellular senescence, disrupted intercellular communication, stem cell depletion, immune system dysfunction, accumulation of toxic metabolites, and dysbiosis. Given the growing interest in the aging area, we propose to add a new hallmark: impaired water homeostasis. This potential hallmark could play a critical role in aging processes and might open new directions for future research in the field. This review enhances our understanding of the physiological aspects of aging in dogs, suggesting new clinical intervention strategies to prevent and control issues that may arise from the pathological degeneration of these hallmarks.

## 1. Multifactorial Nature of Physiological Aging Process

Aging is a complex and multifactorial biological phenomenon impacting all living organisms, starting immediately after birth and continuing throughout life. It can be defined as a progressive and holistic biological decline, resulting in a gradual decrease in the ability to maintain homeostasis in the face of internal physiological and external environmental stresses [1,2]. Aging processes affect biological systems across multiple levels of organization, starting with biochemical changes at the molecular level and cascading through cells, tissues, and organs, ultimately leading to the observable phenotypes in organisms. As organisms age, their physiological responses may become less efficient, resulting in diminished metabolic, immune, and hormonal functions [3]. Furthermore, behavioral flexibility tends to decrease, making adjusting to new situations or acquiring new skills more challenging. Older organisms often face longer recovery times, highlighting their increased disease vulnerability [4,5].

Recent studies in dogs have underscored how age influences physiology and behavior [6,7,8]; therefore, age factors must be considered in research involving dogs at different developmental stages [9]. Although the dog is an excellent model for studying human health span, and the population of aging dogs is increasing in parallel with aging humans, few molecular studies have explored age-related physiological changes in dogs. They suggested developing molecular biomarkers to objectively monitor physiological aging and distinguish it from behavioral aging [10].

A significant body of research, including the Dog Aging Project (DAP) [11], involving approximately 50,000 dogs, has contributed to a deeper understanding of the molecular, physiological, and environmental factors influencing canine aging. The table below summarizes key findings from recent studies, highlighting their objectives, results, and implications for both veterinary and translational human health research (Table 1).

These studies not only illustrate the complexity of aging processes in dogs but also provide a valuable framework for exploring interventions aimed at improving health span and longevity in both species.

McKenzie et al. (2022) [19] identified potential biomarkers useful for tracking physiological aging that could enhance our understanding of how molecular processes correlate with chronological aging in dogs [20,21]. It is estimated that approximately 35% of dogs are older than seven years, a figure steadily increasing due to advances in medical research [22]. Today, phenotypic and behavioral parameters are adopted to assess dog aging; however, these indicators are strongly influenced by breed, weight, and lifestyle [22]. Generally, larger breeds age more quickly, while smaller breeds tend to live longer; for instance, giant breeds like Great Danes have an average lifespan of 5–6 years, whereas smaller breeds like Yorkshire Terriers and Dachshunds can live up to 12 years [23].

**Figure 1:**
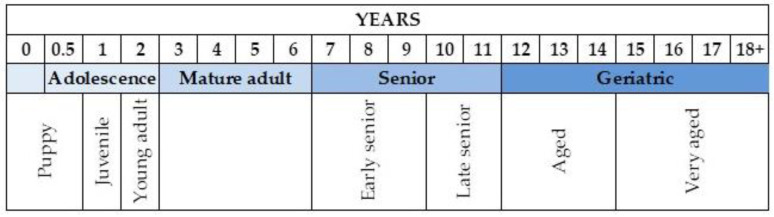
This age-grouping scheme for dogs gives a standard framework for veterinary research and treatment. The canine aging scheme considers cognitive and behavioral development or declines and age-related phenotypic factors based on key differences in longevity with respect to breeds. The categorization includes stages from puppy to geriatric and very old dogs (over 15 years).

Some authors suggest that variability in average life expectancy in dogs makes a clear definition of “geriatric” difficult [24,25]. Nonetheless, they propose that a geriatric pet is typically in the last third of its expected lifespan (Figure 1) [9].

General signs of aging include whitening of the fur, a general decline in coat condition, reduced sensory functions (such as sight and hearing), and lethargy [26]. Behavioral changes, such as increased irritability or nervousness, are also frequently observed. In addition to visible signs, older dogs show metabolic changes that are often undetected by their owners.

Aging in dogs leads to significant changes in the function of key organs, including the kidneys, liver, and heart, with dehydration exacerbating these issues [20,27,28]. As dogs age, kidney function often declines, resulting in reduced urine output quantity and an increased risk of chronic kidney disease (CKD). This decline can lead to dehydration, which further impairs kidney function and accelerates disease progression [29]. Similarly, liver function diminishes with age, compromising the ability to detoxify and process nutrients [30]. Dehydration places additional strain on the liver, reducing its capacity to metabolize substances and maintain fluid balance. Changes in the cardiovascular system also occur with aging; dehydration can lead to reduced blood volume increasing strain on the heart and exacerbating conditions such as heart disease [31,32].

In addition to physiological factors, environmental factors such as stress, exercise, nutrition, and socialization might also influence aging processes in dogs. These factors appear to interact with biological pathways linked to inflammation and oxidative stress [33]. These factors interact with biological processes, particularly through pathways linked to inflammation and oxidative stress, as they are critical in aging. For instance, poor psychological environments and stress have been shown to promote inflammation in aging dogs, contributing to morbidity and cognitive decline. In addition, lifestyle factors such as exercise and nutrition influence the immune system, which tends to weaken with age. Nutrition is particularly crucial for senior pets, as it can help slow down or even prevent some age-related metabolic changes [34,35]. Managing these environmental factors may help mitigate oxidative and inflammatory damage, thus promoting healthier aging in dogs [36].

## 2. Key Drivers of Aging

**Figure 2:**
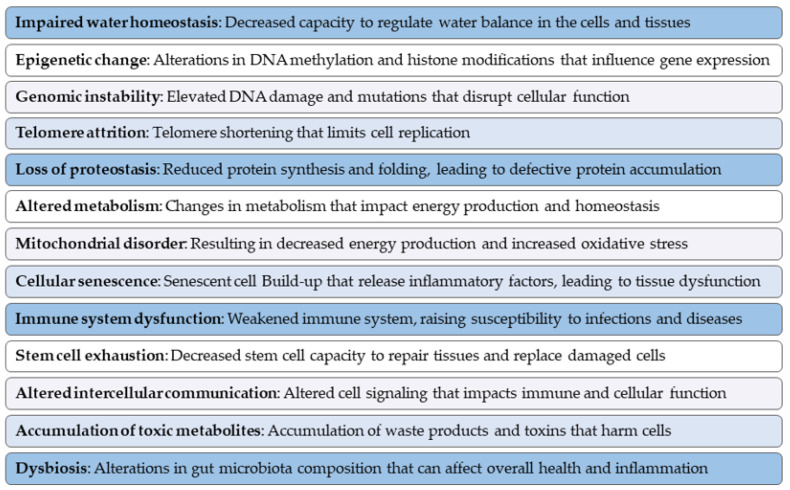
The figure illustrates the key hallmarks of aging in dogs. The concept of impaired water homeostasis is a progressive decline in the body’s ability to retain and properly manage water across cellular and tissue compartments. Impaired water homeostasis accelerates the other hallmarks of aging. This point has not been explored in earlier research on dogs or any other species. Epigenetic changes modify gene expression through changes in DNA methylation and histone modifications, and genomic instability is characterized by increased DNA damage and mutations. Telomere attrition limits cellular replication, while loss of proteostasis leads to defective protein accumulation due to impaired protein synthesis and folding. Altered metabolism affects energy production and homeostasis, and mitochondrial disorder further reduces energy output and increases oxidative stress. Cellular senescence contributes to tissue dysfunction through the accumulation of senescent cells that secrete inflammatory factors. Immune system dysfunction increases susceptibility to infections. Stem cell exhaustion reduces the ability to repair tissues, while altered intercellular communication impairs immune response and cellular function. The accumulation of toxic metabolites leads to cellular damage, and dysbiosis is an alteration of the gut microbiota that leads to inflammation, metabolic disorders, and gastrointestinal diseases.

In earlier research, scientists proposed nine key characteristics, or “hallmarks”, hypothesized to describe aging and its effects on cells and tissues [37]. This concept was developed as a unifying model to organize observed biological changes associated with aging. However, subsequent studies proposed three additional hallmarks, expanding the original framework to twelve fundamental aspects [38]. The twelve hallmarks highlight significant changes associated with aging, i.e., epigenetic alterations, genomic instability, telomere attrition, loss of proteostasis, altered metabolism, mitochondrial dysfunction, cellular senescence, stem cell exhaustion, altered intercellular communication, immune system dysfunction, accumulation of toxic metabolites, and dysbiosis. With the growing body of research on aging, we propose the inclusion of a new hypothetical hallmark: impaired water homeostasis. This important point has not been explored in earlier research on dogs or any other species (Figure 2).

By proposing impaired water homeostasis as a hallmark of aging, we highlight the critical role that maintaining proper hydration plays in preventing or mitigating the physiological decline associated with aging, especially in tissues that are highly dependent on water balance, such as muscles, neurons, and the cardiovascular system. This imbalance could influence overall health and contribute to chronic inflammation [39,40], potentially also negatively affecting the other hallmarks.

In summary, the proposed expansion of the hallmarks of aging provides a more comprehensive understanding of aging. These advancements could offer deeper insights into the mechanisms of aging, opening up new possibilities for interventions and prevention. Listed below are the hallmarks of aging, along with a summary highlighting the key aspects of the discussed topic.

**Figure 3:**
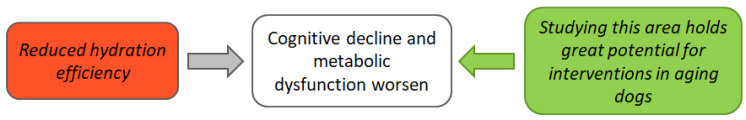
Reduced hydration efficiency worsens aging-related issues, highlighting the potential for targeted dog interventions.

- Impaired water homeostasis (Figure 3).

Aging animals experience changes in body composition [41,42,43], i.e., a decrease in total cell mass, intracellular water, and protein stores, and increase in fat stores [38,44]. As individuals age, the intracellular water content decreases due to the reduced ability of the body to maintain water balance, decreased kidney efficiency, reduced muscle mass, and increased fat tissue, which stores less water. Decreased body water impairs several vital functions. At the cellular level, it reduces volume, disrupting metabolism and nerve transmission [45]. Dehydration compromises nerve transmission by disrupting electrolyte balance and slowing signal propagation. In the elderly, this imbalance can induce cognitive symptoms [46,47]. Dehydration in dogs is known to cause neurological symptoms such as lethargy, disorientation, and reduced mental alertness, resembling signs observed in elderly humans [48]. In senior dogs, cognitive decline—often referred to as “cognitive dysfunction syndrome”—may be exacerbated by dehydration, worsening memory-related symptoms, and confusion [48,49]. Therefore, maintaining proper hydration is crucial for optimal neurological function in dogs as well [50]. Loss of intracellular water decreases the body’s ability to regulate temperature declines, increasing the risk of hyperthermia. The cardiovascular system struggles with reduced plasma volume, leading to a higher heart rate. Digestion slows, potentially causing gastrointestinal issues, while the kidneys become less efficient at eliminating toxins, raising the risk of kidney stones and damage [45].

**Figure 4:**
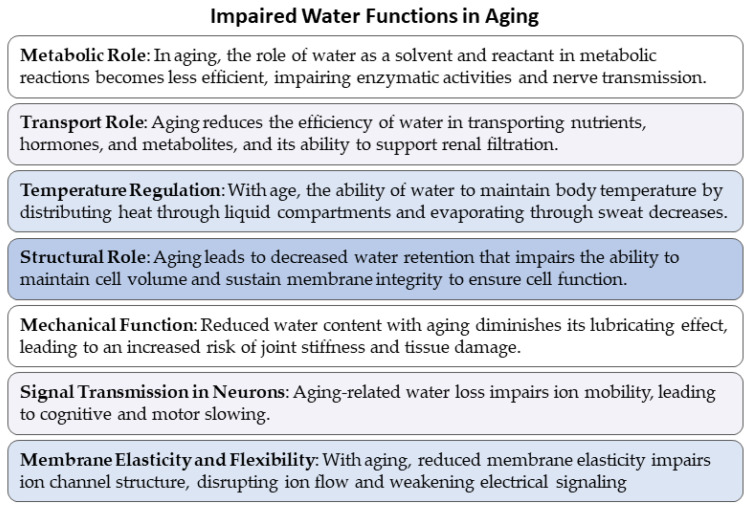
The figure summarizes the seven key roles that water plays in the physiology of aging dogs, particularly as it relates to impaired water homeostasis: metabolic role [53,54], transport role [55], temperature regulation [56], structural role [57], mechanical function [58], signal transmission in neurons [59], and membrane elasticity and flexibility [60]. Each function highlights the loss of a critical aspect of water’s importance in maintaining cellular health, facilitating metabolic processes, ensuring proper signal transmission, and preserving structural and mechanical integrity.

Reduced hydration may indirectly contribute to the decline of the immune system during aging by dehydrating immune cells and impairing the metabolic functions essential for an effective immune response [51,52]. Water serves multiple crucial roles in maintaining physiological balance in dogs throughout their lives, and these roles become even more vital as the body ages. Water plays several crucial roles in maintaining physiological balance in aging dogs, including acting as a metabolic solvent, a transport medium for nutrients and waste, a regulator of body temperature, a structural component for cell volume and membrane integrity, a mechanical lubricant for tissues, a mediator for electrical signal transmission in neurons, and a key element in preserving the elasticity and flexibility of cellular membranes. Understanding these roles is essential for assessing the impact of dehydration and water imbalance on the health of senior dogs (Figure 4).

Metabolically, water functions as both a solvent and a reactive component in various metabolic reactions, facilitating enzyme activities and nerve transmission. As the primary medium for biochemical processes, water ensures that nutrients, hormones, and metabolites are transported efficiently within the bloodstream and supports the elimination of metabolic waste through renal filtration [61]. With aging, dogs face a reduction in both intracellular and extracellular water, impairing these vital functions. Water also plays a key role in regulating body temperature through mechanisms such as sweating and heat distribution within the body’s fluid compartments. As dogs age, this ability diminishes, increasing their vulnerability to heat stress or hyperthermia, particularly during physical exertion or in warm climates. Structurally, water is essential for maintaining cell volume and membrane integrity. It helps preserve the flexibility and functionality of cellular membranes, including those that host ion channels necessary for signal transmission. The elastic nature of hydrated membranes is vital for maintaining proper intercellular communication and for the functioning of proteins embedded within the membrane, such as those responsible for electrical signaling. Moreover, water acts as a mechanical lubricant in various tissues, such as joints, eyes, and mucosal surfaces, protecting them from injury and wear over time [62]. In terms of neurological function, water serves as a mediator for the electrical signaling required for nerve transmission. The movement of ions such as sodium (Na^+^), potassium (K^+^), and chloride (Cl^−^), which are dissolved in water, is essential for generating and propagating action potentials in neurons [63,64,65]. In aged dogs, dehydration leads to electrolyte imbalances that disrupt this ion flow, slowing signal transmission and impairing cognitive functions. This is particularly evident in conditions like canine cognitive dysfunction syndrome (CCDS), where symptoms such as confusion and disorientation can be exacerbated by insufficient hydration. Maintaining proper hydration is thus crucial not only for metabolic and structural reasons but also for sustaining cognitive health and preventing neurological decline in aging dogs [66,67].

**Figure 5:**
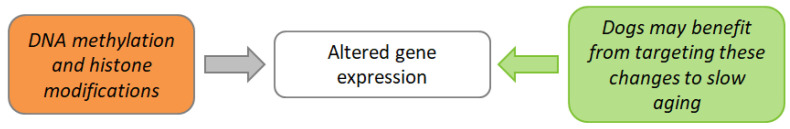
Targeting epigenetic changes like DNA methylation and histone modifications may help slow aging in dogs.

- Epigenetic changes (Figure 5).

Research carried out in 2021 on a cohort of 27,547 dogs belonging to the DAP reported a key concept that older dogs are more affected by unfavorable environmental conditions [68]. Living in a disadvantaged environment, with limited access to resources such as green spaces, clean water, or healthy living conditions, appears to have a greater impact on the health of older dogs compared to younger ones. This effect is similar to what has been observed in humans: elderly individuals living in socioeconomically disadvantaged environments tend to experience worsening health outcomes, exacerbated by pre-existing conditions and a reduced ability to cope with environmental stressors. This reduced capacity, often described as the “decline in adaptability”, reflects the diminished ability of aging organisms to respond effectively to environmental changes and stressors. As organisms age, their physiological responses may become less efficient, leading to decreased metabolic, immune, and hormonal functions, while behavioral flexibility diminishes, making it more challenging to adjust to new situations or acquire new skills. Additionally, older organisms often experience longer recovery times, underscoring their increased disease vulnerability [4,5]. Therefore, this study suggests that the environmental and social context may directly influence health during aging in dogs and humans, highlighting the importance of a favorable environment to improve the quality of life for elderly companion animals [11,68].

Epigenetics, as a field, decodes how cells “utilize” only portions of the genome, enabling them to “interpret” external signals that induce cellular plasticity and adaptability [4,5]. The environmental influence on aging correlates with a promising new mammalian theory that epigenetic modifications may be relevant factors in mammalian aging [69]. Epigenetics concerns reversible heritable modifications regulating gene expression without modifying the DNA sequence. Yang et al. (2023) observed that mammalian aging could be partly driven by epigenetic instability due to DNA damage, particularly double-strand breaks that disrupt chromatin organization [70]. This research also suggests that this loss may be reversible through epigenetic reprogramming, using factors such as OSK (Oct4, Sox2, and Klf4) [70]. In short, this study shows that aging is not exclusively caused by genetic mutations but is driven significantly by epigenetic instability. This loss of information can possibly be manipulated, offering potential pathways to rejuvenate cells and tissues [71].

Epigenetic changes that directly contribute to aging and age-related diseases include DNA methylation and histone modifications [72], alterations in chromatin accessibility [73], loss of histones and heterochromatin [74], aberrant histone modifications, and deregulated expression or activity of microRNAs (miRNAs) [75]. Recent studies have shown that, as in humans, aging in dogs is marked by distinct epigenetic changes [76,77].

Chromatin, which packages DNA around histone proteins, acts as a dynamic interface between genetic information and environmental factors. Gene accessibility depends on chromatin condensation, which is regulated by histone modifications like methylation and acetylation [78]. During aging, histone acetylation decreases, leading to tighter chromatin and reduced gene accessibility. Aging cells also experience chromatin reorganization, including the loss of core histones, changes in histone variants, diminished heterochromatin, defects in nuclear structure, and alterations in histone marks [79].

One of the most studied epigenetic changes in aging dogs is the shift in global DNA methylation patterns, where methylation increases in some regions and decreases in others, affecting gene regulation and contributing to aging processes. DNA methylation assesses the presence of cytosine at the 5′ end of C-G dinucleotides, called CpGs. These dinucleotides are often found near gene promoters and are associated with gene expression levels. The level of DNA methylation at promoter-associated CpGs is generally negatively associated with gene expression, although for some specific genes, the effect is the opposite [80]. DNA methylation profiles have been used as “epigenetic clocks”, biomarkers that predict biological age and health status [69]. Initially designed for humans, epigenetic clocks have been adapted for dogs and other mammals [81]. Thompson et al. (2017) developed an epigenetic clock for dogs and wolves, based on DNA methylation data from blood samples [82]. Their findings reveal that age-related epigenetic changes in canids closely resemble those observed in humans, suggesting an evolutionarily conserved aging mechanism across mammalian species. This epigenetic clock allows the estimation of a dog’s biological age and provides a foundation for further research on aging and its implications for health and longevity in domestic and wild animals [82]. In 2024, Jin et al. built three age prediction models for dogs, incorporating DNA methylation and chromatin accessibility, that revealed age-related genes linked to cognitive decline and provided deeper insights into canine aging [76].

Understanding that aging is driven by epigenetic factors, rather than solely genetic mutations, offers a significant advantage in improving the quality of physiological aging. Unlike genetic mutations, epigenetic changes are reversible, offering opportunities to slow or reverse aging. Therefore, studies in dogs should focus on epigenetic mechanisms, not only for the well-being of the animals but also for their potential implications for human health. The study of miRNAs, which regulate gene expression and influence biological processes such as cell proliferation, differentiation, metabolism, and apoptosis, is suggested. These small RNA molecules inhibit translation through partial binding to target mRNAs or by silencing gene expression via cleavage of target genes. MiRNAs are increasingly recognized for their role in aging and age-related diseases, modulating pathways like cellular senescence, tissue repair, and inflammation [4,75,83,84]. Research suggests that targeting miRNAs could lead to therapeutic interventions for age-related conditions in dogs and humans [85,86].

**Figure 6:**
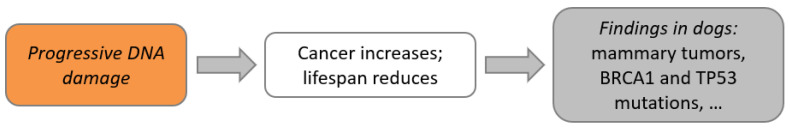
Progressive DNA damage in dogs leads to cancer and reduced lifespan.

- Genomic instability (Figure 6).

As cells age, the nucleus—the command center of the cell housing the genetic material—undergoes relevant changes in the genome that affect how the cell functions. It is now thought that genomic instability could be a fundamental cause, rather than a consequence, of aging [87]. Over time, DNA integrity and stability are constantly challenged by external and internal factors. External factors include physical, chemical, and biological agents, while internal factors encompass DNA replication errors, spontaneous hydrolytic reactions, and oxidative damage [88]. These damages may result in genome instability characterized by a tendency to undergo permanent mutations, and inheritable changes to the DNA sequence, such as base substitutions, insertions, deletions, chromosomal abnormalities, telomere shortening, and gene disruption by viruses or transposon integrations [87]. To prevent or correct these damages, mammalian cells possess DNA repair mechanisms whose efficacy declines with age, leading to genomic instability when the damage exceeds the repair capacity [89]. This may impact cellular function and overall health, potentially leading to age-related diseases and influencing lifespan.

At present, there is limited research on the role of DNA damage or repair defects in dog aging. However, given the similarities between the genomes and DNA repair systems in dogs and other mammals such as humans, where the role of DNA damage in aging and the development of age-related neoplasia is well studied, DNA damage is probably an important factor in canine aging as well [89,90,91]. Multiple studies analyzing different forms of canine cancer have revealed alterations in the DNA repair mechanism similar to those found in human cancers [92,93,94]. For instance, Golden Retriever lymphomas have shown a reduced capacity for DNA damage response [92], while decreased serine/threonine kinase ATM gene expression has been observed in canine mammary tumors [93].

Genetic variations in breast cancer 1 (BRCA1) and tumor protein p53 (TP53) genes have also been linked to several types of canine cancers [95,96].

Oxidative stress (OS) is a major cause of intrinsic DNA damage. OS in the cell is mainly caused by chemical interactions between cellular constituents and reactive oxygen species (ROS), which chemically act as free radicals characterized by high oxidative activity [97]. There are many sources of ROS: mitochondrial respiration, ionizing radiation, and the activity of specific enzymes such as NADPH oxidase and double oxidase (DUOX) [98]. These agents affecting DNA can have a relevant impact on cellular function. The mechanism of action of ROS in the nucleus is the oxidation of the guanine base of DNA to form 8-hydroxydeoxyguanosine (8-OHdG). 8-OHdG can pair with adenine instead of cytosine during DNA replication, resulting in mutations in the DNA sequence [99]. Research has established that oxidative DNA lesions accumulate progressively with age. Several studies have confirmed that elderly dogs have increased levels of oxidative damage in the brain, as indicated by the accumulation of carbonyl groups [100], lipofuscin [101], 4-hydroxynonenal [102], and malondialdehyde [103] in neuronal tissue.

**Figure 7:**
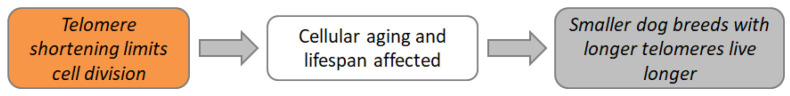
Telomere shortening influences aging, with smaller dogs having longer telomeres and a longer lifespan.

- Telomere attrition (Figure 7).

Genomic stability systems include mechanisms to maintain telomeres’ proper length and functionality as protective caps at chromosome ends [44]. In mammals, telomeres consist of TTAGGG sequence repeats and associated proteins, crucial for maintaining genomic integrity by shielding chromosome ends from recombination, fusion, and DNA damage recognition [104]. Telomeres shorten as cells divide, leading to DNA damage, cell cycle arrest, and pro-inflammatory factor expression, which is associated with aging. Telomere shortening is a hallmark of aging, and there is evidence suggesting that accelerated aging may be linked to telomerase deficiency or induced telomere attrition [105]. Domestic dogs exhibit telomere biology similar to humans, with comparable telomere length, telomere shortening, and a lack of telomerase activity in somatic cells [106]. Research by Fick et al. using peripheral blood mononuclear cells (PBMCs) found that telomere length strongly correlates with average lifespan across different dog breeds, suggesting a potential role in the longevity of these animals [107]. Dogs lose telomeric DNA approximately 10 times faster than humans, reflecting the ratio of average lifespans between these species. Breeds with shorter average telomere lengths tend to have a higher probability of death from cardiovascular disease, a correlation previously observed with shorter telomere length in humans [107].

Sirtuins, a class of NAD+-dependent histone deacetylases with a conserved central catalytic domain, appear to play a key role in regulating genomic stability and DNA repair. In several organisms, the overexpression of specific sirtuins has been linked to extended lifespan [108]. Mammals possess seven sirtuins, SIRT1-7, which differ in catalytic activities, subcellular localization, protein targets, and biological functions. Suppression of cellular senescence by sirtuins is mediated primarily by preventing telomere attrition, maintaining genome integrity, and promoting DNA damage repair [109,110]. SIRT1 and SIRT6 play a crucial role in regulating the expression of telomere reverse transcriptase, which is essential for telomere elongation. They also deacetylate histone 3 at lysine 9 (H3K9) and lysine 56 (H3K56), thereby preserving telomeric integrity. Furthermore, SIRT1 and SIRT6 are recruited to sites of DNA damage, where they facilitate DNA repair by deacetylating key repair proteins, including poly (ADP-ribose) polymerase (PARP)-1, Ku70, NBS, and Werner (WRN) helicase [111]. In mammals, SIRT levels typically decline with age, which is correlated with increased DNA damage. Sirtuins remain relatively unexplored in the context of canine aging [44,89]. Research using primary fibroblast cells from both young and elderly dogs of large and small breeds shows that SIRT1 levels decrease with age in both breed sizes [112]. Additionally, DNA damage is more significant in primary fibroblasts from older large-breed dogs [90,112]. These findings suggest that small-breed dogs may possess distinct DNA repair mechanisms compared to large-breed dogs.

**Figure 8:**
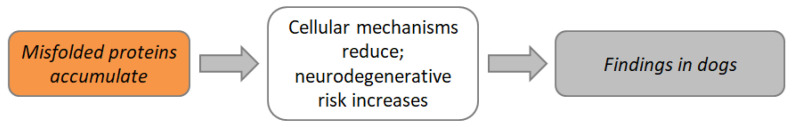
Accumulation of misfolded proteins disrupts cellular mechanisms and elevates the risk of neurodegenerative diseases in aging dogs.

- Loss of proteostasis (Figure 8).

Mammalian cells maintain proteome balance through tightly regulated processes such as protein synthesis, folding, transport, post-translational modification, and degradation [113]. The loss of protein homeostasis (proteostasis) is a key feature of aging and may drive it [38]. The proteostasis network (PN), a collection of systems responsible for the synthesis, folding, disaggregation, and degradation of proteins, preserves proteome stability by preventing the accumulation of misfolded or damaged proteins. This network includes Heat-shock proteins (HSPs), chaperones, the ubiquitin–proteasome system (UPS), and the autophagy lysosomal pathway (ALP). HSPs can refold misfolded proteins, while chaperones like Hsc70, the UPS, and APL target irreparable proteins for degradation. With age, cells accumulate misfolded proteins due to declining proteostasis efficiency, leading to reduced viability and the onset of protein-misfolding disorders [44]. Older cells exhibit more oxidative protein damage (i.e., carbonylation, oxidized methionine, glycation) and less active enzymes, contributing to protein aggregation and tissue decline [114]. Misfolded proteins, particularly those with exposed hydrophobic regions, tend to form toxic aggregates, disrupting membranes and cellular components. These aggregates overwhelm proteostasis systems, further impairing the cell’s ability to maintain proteome integrity, even in small amounts, severely affecting cellular function [115].

Molecular chaperones are essential for maintaining protein balance by ensuring correct protein folding after synthesis. Chaperones are classified into families such as Hsp70, Hsp90, DNAJ/HSP40, chaperonin/Hsp60, and small Hsp (sHsp). They function independently or in combination with cochaperones to manage protein folding, disaggregation, degradation, and intracellular trafficking [116]. Chaperones like the Hsp70 family stabilize intermediate folding states and use ATP to drive proper folding. These proteins play a key role in identifying newly synthesized or damaged polypeptides, facilitating their proper folding, or directing irreparable proteins toward ubiquitination and eventual degradation by the proteasome. Research on aging Labrador retrievers revealed a decline in serum levels of Hsp70 with age, suggesting a reduced capacity for proper protein refolding in older dogs [117]. However, comparative studies across multiple dog breeds are still lacking, limiting broader insights into how aging impacts proteostasis in canines [44]. In contrast, a study by Ghi et al. (2009) on the hippocampi of dogs from various breeds reported an age-related increase in Hsp90 levels, which could reflect a compensatory response to accumulated protein damage [118].

When proteins become misfolded or aggregated and cannot be repaired, molecular chaperones direct them to degradation pathways, primarily through the UPS [119]. The UPS marks proteins for destruction by the proteasome, a large proteolytic complex consisting of a 19S regulatory cap and a 20S catalytic core. This process begins by attaching polyubiquitin chains to target proteins [120]. Once a protein is tagged with ubiquitin, the 19S regulatory particle recognizes the ubiquitylated substrate, removes the ubiquitin chains, and unfolds the protein to facilitate its entry into the 20S core. In the core, the protein is rapidly cleaved into smaller peptides, completing the degradation process [119]. Proteasome function declines with aging and senescence in mammalian tissues due to reduced proteasome subunit expression, decreased activity, and subunit modifications. Protein aggregates also impair proteasomes, creating a harmful feedback loop where accumulated misfolded proteins further inhibit proteasome function, worsening protein damage and aggregation [121,122]. In dogs, research on UPS activity during aging has focused on the brain, revealing some notable findings [121]. Data suggest that aging leads to a decline in the basal expression of several key components of protein quality control systems in the canine hippocampus, a critical area for cognition and memory. Specifically, reductions in Psmd4 (Proteasome 26S Subunit Ubiquitin Receptor, Non-ATPase 4), Psmb8 (Proteasome 20S Subunit Beta 8), and egr1 (Early growth response protein 1) expression, coupled with increases in Psmb9 and Hsp90 levels, are consistent with a deterioration in UPS function with age [118]. Additionally, an increase in the density of ubiquitinated proteins in the aging canine brain has been observed, indicating a decreased rate of proteolysis for damaged proteins [121].

The ALP, also known as macroautophagy, is a catabolic cellular process that degrades cellular components—proteins, lipids, organelles, and even pathogens—to provide metabolites and energy to cells [89]. This highly conserved process plays a central role in maintaining cellular homeostasis by regulating metabolism, enhancing pathogen resistance, controlling inflammation, clearing accumulated macromolecular debris, and facilitating programmed cell death [89]. Macroautophagy starts by forming double-membrane vesicles called autophagophores, which encapsulate the targeted cytoplasmic materials and fuse with lysosomes for degradation and recycling [123]. The discovery of “autophagy-related genes” (Atgs) in yeast and their subsequent identification in mammals shows how autophagy is regulated. These Atgs are responsible for the initiation and maturation of autophagosomes. The main regulator of autophagy in mammals is the mammalian target of the Rapamycin (mTOR) complex, which suppresses autophagy by phosphorylating and inhibiting the upstream serine-threonine kinase ULK1, a key activator of macroautophagy. When mTOR activity is inhibited, autophagy is induced, promoting cellular survival and repair [122]. Autophagy plays a critical role in regulating aging, with impaired macroautophagy possibly contributing to accelerated aging, while its restoration could potentially extend life expectancy [122]. As organisms age, the function of lysosomes deteriorates, leading to the accumulation of damaged and misfolded proteins within cells [124]. In mammals, aging is associated with a reduced capacity to clear autophagosomes and a diminished response to hormonal signals that stimulate autophagy [114]. This reduction in autophagic efficiency is linked to the development of age-related neurodegenerative diseases, as observed in mice [125,126] and humans [127].

Dogs provide a unique model for studying the relationship between autophagy, aging, and disease. Several canine hereditary diseases have been linked to mutations in Atgs [128,129,130]. Moreover, the emerging field of dog aging research provides an opportunity to better understand the broader implications of autophagy in lifespan regulation. For instance, the US FDA approved Rapamycin, a naturally occurring macrolide compound acting as an allosteric inhibitor of mTORC, for treatment in cancer and transplant patients [131,132]. Studies in several model organisms have demonstrated that Rapamycin treatment extends lifespan and improves health span, largely through its effects on autophagy [124]. The DAP is currently exploring the potential benefits of mTOR inhibition on canine health and longevity. This research aims to determine whether Rapamycin can improve the health span in dogs, potentially providing valuable insights into how autophagy modulation could benefit both canine and human aging [11]. Studying the effects of autophagy-related therapies in dogs could lead researchers to suggest new ways to promote healthy aging across species.

**Figure 9:**
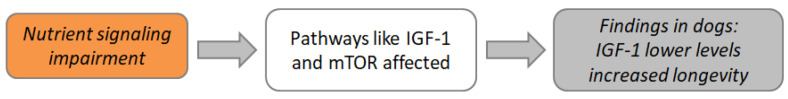
Impaired nutrient signaling impacts longevity, with lower IGF-1 levels associated with increased lifespan in dogs.

- Altered metabolism (Figure 9).

Metabolic homeostasis is a fundamental component of cellular and organismic homeostasis, as nutrients are crucial for biological processes. Cellular pathways that monitor nutrient availability and energy status interact with hormones and growth factor signals to regulate metabolic homeostasis in a coordinated manner. Aging leads to a gradual decline in these regulatory functions, creating a bidirectional relationship where aging impairs metabolic pathways, and metabolic dysregulation, in turn, is thought to accelerate the aging process [133]. Disruptions in nutrient signaling systems are a key factor in the loss of metabolic homeostasis, contributing significantly to aging and age-related diseases. Insulin/insulin-like growth factor-1 (IGF-1) signaling, mTOR, AMP-activated protein kinase (AMPK), and sirtuins are the most extensively studied nutrient-sensing pathways that have been shown to regulate lifespan in various model organisms [134,135,136]. These pathways play an essential role in regulating cellular processes like protein synthesis, autophagy, metabolism, oxidative stress, and immunity. Under conditions of nutrient availability and low stress, they facilitate growth and reproduction. However, when nutrients are scarce and stress levels rise, these pathways shift to conserve energy [137].

IGF-1 signaling is the metabolic pathway most characterized to be connected with aging and age-related diseases [138]. Research has shown that mutations or downregulation of key components in this pathway are associated with increased longevity in worms [139], insects [140], and mammals [141]. It is closely tied to the IGF-1 pathway’s role in regulating metabolism, growth, and cellular maintenance [142]. In response to nutrient availability and/or sensory signals, insulin and insulin-like peptide ligands activate the IGF-1 pathway, which is part of the somatotropic axis consisting of the growth hormone (GH), IGF-1, their receptors, and the related downstream cascade [137]. In mammals, the GH is released from the anterior pituitary gland in response to hypothalamic GH-releasing hormone (GHRH), insulin-induced hypoglycemia, and physical activity [138]. GH then regulates levels of IGF-1, predominantly produced by liver cells, by activating IGF-1 gene expression. IGF-1-mediated feedback on the hypothalamus suppresses GHRH expression, completing part of the somatotropic axis regulatory circuit [143]. IGF-1 binding to its receptor (IGF-1R) in the plasma membrane triggers downstream signaling through pathways like PI3K/AKT, mTOR, and RAS/MAPK, all of which contribute to controlling metabolism and growth [144,145].

The normal aging process is characterized by a decline in circulating levels of IGF-1 and GH and reduced IGF-1 pathway activity, which have been linked to increased longevity. Interestingly, genetic mutations impairing GH, IGF-1 receptors, insulin receptors, or downstream effectors such as AKT, mTOR, and FOXO are associated with extended lifespan in humans and model organisms [37]. These seemingly paradoxical findings may be explained by reduced IGF-1 signaling acting as a protective mechanism, limiting cell growth and metabolism in response to age-related cellular damage [89,146]. According to this perspective, organisms with chronically lower IGF-1 signaling may live longer due to lower rates of cellular growth and reduced damage over time. In dogs, as in humans and other mammals, IGF-1 levels decline with age [147]. Larger dogs, which tend to have higher GH and IGF-1 levels, generally have shorter lifespans than smaller breeds [147,148,149]. This suggests a potential correlation between IGF-1 signaling and canine lifespan, with smaller breeds exhibiting reduced IGF-1 levels and longer life expectancy [147].

mTOR is a highly conserved serine/threonine kinase acting as a critical regulator of lifespan across eukaryotes by sensing and responding to nutrient availability and growth signals. In mammals, mTOR operates through two multiprotein complexes, mTOR complex 1 (mTORC1) and mTOR complex 2 (mTORC2) [150]. mTOR functions as an energy rheostat, sensing various extracellular stimuli and integrating intracellular input from IGF-1 and other growth factors, cellular energy sensor AMPK, amino acids, lipids, cholesterol, and oxygen levels [150,151]. mTORC1, along with its downstream effectors ribosomal protein S6 kinase β1 (S6K1) and eukaryotic translation initiation factor 4E-binding protein 1 (4E-BP), is essential for cell survival, driving cell growth and protein synthesis based on nutrient availability. When nutrients are abundant, mTORC1 promotes anabolic processes, including protein, nucleotide, and lipid biosynthesis, while inhibiting catabolic processes such as autophagy and lysosomal biogenesis. Consequently, mTORC1 is rapidly inactivated under nutrient stress—such as decreased intracellular ATP or amino acid availability—or in response to various cellular damages, halting cell growth [152].

As aging progresses, mTORC1 activity increases, driving intense anabolic processes that accelerate aging and the onset of age-related diseases [153]. In a pilot study involving 24 middle-aged companion dogs, treatment with Rapamycin (0.1 mg/kg, 3 times/week) for 10 weeks showed improvements in cardiac function, specifically enhanced diastolic and systolic functions [18]. This study aligns with findings from mouse studies where Rapamycin treatment, initiated in middle to old age, rejuvenated several cardiac aging phenotypes, highlighting the critical role of mTORC1 in cardiac aging [154]. A larger study is currently underway, aiming to further evaluate Rapamycin’s long-term effects on aging in dogs [12].

AMPK and sirtuins, the two other key nutrient sensors, are in contrast to IGF-1 signaling and mTOR. While IGF-1 and mTOR respond to nutrient abundance by promoting anabolism, AMPK and sirtuins signal nutrient scarcity and drive catabolism. As a result, their activation supports healthy aging by encouraging metabolic processes associated with longevity. AMPK is activated in response to cellular energy shortages and helps restore energy balance by promoting catabolic pathways that generate ATP (i.e., glucose uptake, fatty acid oxidation) while inhibiting anabolic pathways that consume ATP (i.e., synthesis of cholesterol, fatty acids, proteins, and glycogen) [155]. AMPK activation occurs through direct binding with AMP or elevated levels of AMP, ADP, and calcium, and is regulated by upstream kinases like liver kinase B1 (LKB1) and calcium/calmodulin-dependent protein kinase kinase 2 (CAMKK2), which respond to increases in intracellular calcium levels [137]. AMPK activation can regulate the function of various transcription factors directly or indirectly. It can stimulate the sirtuin 1 (SIRT1) pathway, which in turn co-activates cAMP response element-binding protein (CREB), CREB-regulated transcription coactivator-2 (CRTC2), and the FOXO pathways, enhancing stress resistance and promoting longevity [155]. Additionally, AMPK contributes to lifespan extension by modulating inflammation and inhibiting the pro-inflammatory transcription factor NF-κB [137]. Moreover, AMPK negatively regulates mTOR by targeting TSC2 and the mTORC1 component raptor [156]. Aging is associated with a gradual decline in AMPK activity, which contributes to various age-related metabolic disorders such as cardiovascular diseases, neurodegenerative disorders, and metabolic syndromes [157]. Metformin, a widely used diabetes medication known to activate AMPK, has gained attention for its potential aging effects [158]. Beyond its glucose-lowering effects, metformin exhibits a range of anti-aging benefits at both the cellular and organismal levels, closely linked to improvements in key aging hallmarks such as inflammation, autophagy, and cellular senescence [159]. Recent studies in dogs have demonstrated that metformin provides protective effects against induced heart failure [160], while there are still limited data regarding the use of metformin to treat diabetes in dogs [161].

The sirtuin family, especially SIRT1, is gaining significant attention for its potential link to longevity, primarily due to its critical role in cellular metabolic regulation. SIRT1 influences metabolic pathways by regulating gluconeogenesis and glycolysis, particularly through the nuclear localization of PGC-1α. It promotes gluconeogenesis and fatty acid oxidation while inhibiting glycolysis [137]. There is a vital connection between nutrient and energy sensors, where sirtuins interact with all major conserved longevity pathways, including AMPK, IGF-1, mTOR, and the transcription factor FOXO. Notably, AMPK and sirtuins act as energy sensors in a manner that contrasts with the nutrient-sensing function of mTOR. AMPK and sirtuins drive together the catabolism of metabolites while suppressing their anabolism [109]. IGF-1, mTOR, AMPK, and sirtuin pathways are interconnected to precisely fine-tune and regulate metabolic responses according to the cell’s energy status, nutrient availability, and hormone or growth factor signaling.

**Figure 10:**
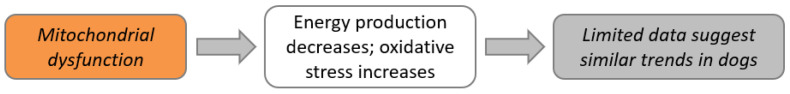
Mitochondrial dysfunction reduces energy and increases oxidative stress, with similar patterns seen in dogs.

- Mitochondrial disorder (Figure 10)

Mitochondrial dysfunction is increasingly recognized as a critical factor in aging and the onset of age-related diseases. Mitochondria are the primary energy sources within cells, producing ATP through oxidative phosphorylation (OXPHOS) [162]. Mitochondria play an essential role in regulating metabolism and cellular homeostasis, contributing to bioenergetics, ROS production, anabolic and catabolic processes, calcium and iron balance, apoptosis, and signaling. During aging, mitochondria become larger and fewer in number, their respiratory activity declines, and mitochondrial DNA damage increases. Maintaining cellular homeostasis requires a well-coordinated balance between the production of new mitochondria and the removal of damaged mitochondria via mitophagy. This balance is essential for maintaining homeostasis and promoting longevity [163].

Mitochondria generate approximately 90% of cellular ROS, mainly by the loss of electrons from complex I and complex III of the electron transport chain, leading to the partial reduction of molecular oxygen (O_2_) to form superoxide. Superoxide is then rapidly dismutated to hydrogen peroxide by two dismutases, superoxide dismutase 2 (SOD2) in the mitochondrial matrix and superoxide dismutase 1 (SOD1) in the mitochondrial intermembrane space [164]. Both superoxide and hydrogen peroxide generated in this process can be considered mitochondrial ROS [165]. At low levels, ROS are essential for gene regulation, cell signaling, and apoptosis, while excessive ROS production can overwhelm the cell’s antioxidant defenses, leading to OS. This OS causes oxidative damage that further degrades mitochondria and causes widespread cellular damage affecting lipids, DNA, and proteins, ultimately inducing senescence or cell death [165]. Cells contain antioxidant systems to combat the damage caused by ROS production, including enzymatic antioxidants such as glutathione peroxidase (GPx), superoxide dismutase (SOD) and catalase (CAT). These molecules work by catalyzing the oxidation of biologically less harmful molecules. Other antioxidant molecules, such as vitamins E and C, act as chain-breaking antioxidants; they seek out ROS, remove them once they are formed, and further block the propagation of peroxidation [166]. Although cells have these antioxidant systems, mitochondrial ROS production increases with age, which might lead to a corresponding increase in oxidative damage that affects lipids and DNA [167]. The first is lipid peroxidation (LPO), in which a stray electron travels across membranes, altering lipid composition and impairing cellular function. The second is the accumulation of mutations in DNA, which can lead to diseases such as cancer [112]. The OS theory of aging has become one of the currently accepted theories of biological aging because it successfully links the balance between the accumulation of cellular damage and the concentration of pro-oxidants over time [168].

Several studies on dogs have investigated mitochondrial ROS production and aging. In Labrador retrievers, DNA damage increases with age despite unchanged total antioxidant potential [169], while in beagles, age-related increases in lipid peroxidation and protein carbonyls, coupled with decreased glutathione function, suggest that diminished antioxidant defenses contribute to damage accumulation [103]. Leukocytes in beagles show increased complex III activity and decreased DNA and protein damage [170]. Additionally, lipid peroxidation levels in the blood of mixed-breed dogs of similar age and size showed higher levels in males than females [171]. In vitro studies in dogs using primary canine dermal fibroblasts revealed differences in mitochondrial function and oxidative metabolism between short-lived (large) and long-lived (small) dog breeds [172]. The study found that cells from long-lived breeds exhibit less electron escape, deeper respiration, and higher respiratory capacity, indicating a potential role for mitochondria in determining life span in dogs [172].

**Figure 11:**
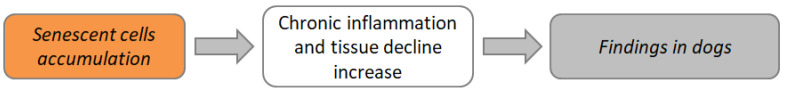
An increase in senescent cells contributes to chronic inflammation and tissue damage, as found in dogs.

- Cellular senescence (Figure 11).

Senescence is a cellular program that triggers permanent cell cycle arrest, accompanied by distinct phenotypic changes such as chromatin remodeling, metabolic reprogramming, enhanced autophagy, and activation of a complex pro-inflammatory secretome. While senescence plays essential roles in normal development, tissue homeostasis, and tumor suppression, it is also a key contributor to age-related diseases [173].

Cellular senescence can be divided into replicative and stress-induced senescence [174]. Telomeres shorten with each cell division, and when they reach a critical length, the cell enters senescence to prevent genomic instability, tumors, and DNA damage whose response is mediated by proteins such as p53 and p21 that arrest the cell cycle and prevent proliferation of damaged cells. On the other hand, stress-induced or secondary senescence is triggered by various factors. These factors do not result directly from cell arrest processes but stem from intercellular communication and molecules released by senescent cells [175]. Such factors include pro-inflammatory molecules, ROS, the NF-kB pathway, and Wnt signaling dysfunction [176]. This form of senescence is induced by extracellular mediators of inflammation and fibrosis (CCL2, IL-1β, IL-6, IL-8, and TGF-β) [177]. In aging, the contribution of stress-induced senescence is thought to be more relevant because the accumulation of cellular damage is more frequent than telomere shortening, which is typical of highly proliferative cells [178].

To date, several markers have been identified in mammals to measure biologically cellular senescence, with DNA damage being one of the most used [44]. The accumulation of DNA damage in aged tissues has been associated with the expression of senescence-associated enzyme β-galactosidase (SA-β-gal) [179]. This phenomenon is due to increased lysosomal activity in senescent cells, possibly caused by a higher number of lysosomes during senescence [180]. Studies show that some human senescent fibroblasts and keratinocytes express β-galactosidase during senescence in culture [181].

The INK4a/ARF locus and the p53 gene are key elements in controlling cellular senescence, regulated by genetic and epigenetic factors. In proliferating cells, the INK4a/ARF locus is normally inactivated by epigenetic modifications such as methylation of lysine 27 of histone H3 (H3K27) and ubiquitination of lysine 119 of histone H2A mediated by the Polycomb complex. However, under epigenetic stress, these modifications are altered, promoting activation of the INK4a/ARF locus and thus contributing to senescence. A meta-analysis of more than 300 genome-wide association studies (GWASs) identified the INK4a/ARF locus as strongly associated with many age-related diseases, including cardiovascular disease, diabetes, glaucoma, and Alzheimer’s disease [175].

The p16 INK4a/Rb and p19 ARF/p53 pathways are critical to this senescent response, as their expression increases with age in various tissues and species, suggesting they could serve as key markers of physiological aging [38]. Chromatin remodeling is crucial during senescence, as it blocks the transcription of pro-proliferative genes, preventing cell replication. An example of this phenomenon is chromatin decompression induced by histone deacetylase inhibitors (HDACis), which increase histone acetylation. It makes the chromatin structure more open and activates the expression of genes such as p21, which is responsible for cell cycle arrest. If cell damage is persistent, p16 INK4a/Rb is also activated through mitochondrial dysfunction mediated by the p38-MAPK pathway and the production of ROS, which contribute to the maintenance of senescence [175].

During senescence, DNA damage triggers sustained activation of p53, which promotes the transcription of the cyclin-dependent kinase inhibitor p21. This, in turn, inhibits CDK4/6 activity, leading to the hypophosphorylation of Rb and subsequent cell cycle arrest. Due to the central role of p53, additional regulatory mechanisms are in place. For instance, ARF, a product of the INK4a/ARF locus, sequesters the ubiquitin ligase MDM2, stabilizing p53 levels [182]. Additionally, recent findings have highlighted the role of FOXO4, a transcription factor involved in aging, in regulating p53 localization and transcriptional activity during senescence [173].

Another hallmark of senescence is the formation of senescence-associated heterochromatin foci (SAHF), regions of highly condensed chromatin that sequester and silence proliferation-promoting genes such as E2F and cyclin A. These foci help block the cell cycle, thereby reducing the risk of malignant transformation and maintaining the senescent state in the long term [183].

**Figure 12:**
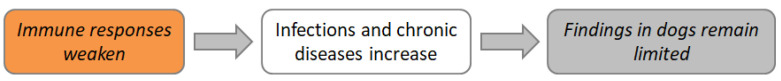
Reduced immune function increases the risk of infections and chronic diseases, though data in dogs are limited.

- Immune system dysfunction (Figure 12).

Senescent cells, which accumulate in various tissues with aging, secrete a range of pro-inflammatory cytokines, chemokines, and other factors collectively known as senescence-associated secretory phenotype (SASP). SASPs can have harmful effects on neighboring cells, promoting tissue dysfunction and inflammation, which may further exacerbate age-related diseases such as atherosclerosis, diabetes, and neurodegenerative diseases [184].

This secretory profile contributes to local and systemic inflammation, playing a role in the phenomenon of “inflammaging”, a chronic inflammation with elevated IL-1, IL-6, IL-8, and TNF-α associated with aging. It results in a reduced ability of the immune system to respond effectively to infection and disease. Chronic inflammation is common in aging dogs, with increased inflammatory cytokines contributing to age-related diseases such as arthritis and kidney disease [117,185].

As individuals age, there is a decline in the production of T and B lymphocytes, primarily due to thymic atrophy and the inefficiency of hematopoietic stem cells. Specifically, older adults show a compromised response to antigens [186]. The decrease in immune cell production suggests that aging dogs experience weakened immune responses, likely due to reduced lymphocyte production and declining thymic function [186]. Altered T-cell quantity and quality can lead to a weakened response to vaccines. This results in a diminished protective capacity from the immune response elicited by vaccinations. Incorporating adjuvants in vaccines can help enhance these immune responses [187].

Presently, measuring the levels of TNF-α, IL-6, and IL-1 can provide valuable insights into inflammatory states that are associated with frailty, alterations in immune function, functional decline, and mortality linked to the phenomenon of inflammaging [188,189].

**Figure 13:**
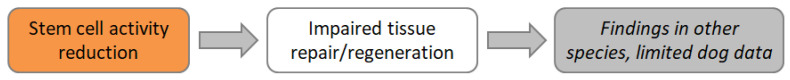
Decreased stem cell activity over time affects tissue repair and regeneration, with limited evidence in dogs.

- Stem cell exhaustion (Figure 13)

Aging is linked to a decline in tissue renewal and impaired repair after injury. Stem cells, which are undifferentiated cells able to self-renew, regenerate, and differentiate into specific cell types, play a key role in maintaining tissues in both developing and adult organisms [190]. The depletion of hematopoietic and mesenchymal stem cells slows tissue repair and disrupts homeostasis. As organisms age, the stem cell pool decreases, and the remaining cells lose their ability to proliferate and differentiate, contributing to tissue aging, a process observed in humans and dogs [38]. Understanding these changes could be crucial for developing therapies for age-related conditions [191,192]. Muscle aging, which leads to the loss of mass and function, is particularly relevant in this context. Muscle tissue, being multinucleated and post-mitotic (meaning that once its cells are mature, they no longer divide), relies on hypertrophic growth (the enlargement of existing cells) for postnatal growth. This process depends on the activation of satellite cells, a subpopulation of mesenchymal stem cells (MSCs) located in the basal membrane of muscle fibers. The activation of satellite cells is influenced by the expression of IGF-1, a growth factor that stimulates stem cell proliferation and muscle growth [193].

Stem cells and progenitor cells, as well as non-stem cells, are all subject to the same hallmarks of aging. Indeed, studies have shown a general decline in cell cycle activity of hematopoietic stem cells (HSCs), with older HSCs undergoing fewer cell divisions than young HSCs. This phenomenon has been associated with the accumulation of DNA damage, overexpression of cell cycle inhibitory proteins such as p16INK4a, and telomere shortening [37]. The general strategy to counteract the age-related decline in stem cell function is based on the concept of “cellular reprogramming”. Rejuvenation of resident stem cells through genetic and epigenetic reprogramming is emerging as an approach to extend active longevity and improve quality of life during aging [194]. In veterinary medicine, stem cell therapy has been explored as a potential treatment for a wide range of age-related diseases, including dermatological, dental, endocrine, neurological, muscular, respiratory, urinary, and gastrointestinal diseases [190].

In the context of canine and human aging, recent studies have focused primarily on mesenchymal stem cells (MSCs), which have demonstrated great therapeutic potential for tissue regeneration [195,196]. MSCs are found in the connective tissue or stroma surrounding organs and can be easily isolated from different tissues, particularly bone marrow and adipose tissue in companion animals. They can differentiate into various cell types and exhibit immunological properties, including anti-inflammatory, immunoregulatory, and immunosuppressive effects. These characteristics make MSCs potential immune-tolerant agents and effective in treating various conditions, positioning them as a promising option for regenerative medicine [197].

Stem cell therapy has shown promise for treating cognitive dysfunction syndrome (CDS), a common condition in senior dogs marked by cognitive decline, memory loss, and behavioral changes [190]. In a study by Valenzuela et al. [198], injecting autologous skin-derived neural precursor cells into the hippocampus of dogs with CDS resulted in improvements or complete reversal of brain function, suggesting that stem cells can differentiate into neurons and promote neurogenesis [198]. Myxomatous mitral valvular disease (MMVD), the most common heart disease in dogs, is also considered age-related [199]. While stem cell therapy has been explored for treating MMVD and congestive heart failure (CHF), results have been mixed. A study by Yang et al. found that intravenous infusion of allogeneic umbilical tissue-derived MSCs was found to be safe but did not show significant therapeutic effects compared to autologous serum therapy [200]. In contrast, another study reported that administering stem cells improved heart function and quality of life in elderly dogs with MMVD [201].

**Figure 14:**
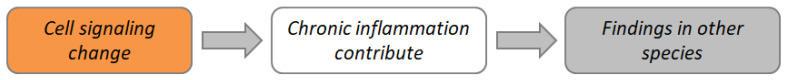
Aging in mammals alters cell signaling, driving chronic inflammation and related pathologies.

- Altered intercellular communication (Figure 14).

Cells are constantly exposed to various external signals, some originating from nearby cells and others transported by the blood from distant tissues. This signaling between cells is known as classical intercellular communication. However, there are also lesser-known forms of communication called non-classical intercellular communication [180]. Non-classical communication involves mechanisms that do not fall under the traditional endocrine, autocrine, or paracrine models. These include communication mediated by exosomes and extracellular vesicles, gap junction communication, and mechanical communication, which does not involve chemical molecules but physical forces, and juxtacrine communication, characterized by direct ligand–receptor binding between adjacent cells [202].

During mammalian aging, classical intercellular communication undergoes various modifications. Paracrine communication in old age is particularly associated with inflammaging and becomes predominant [89]. In aging, paracrine renin–angiotensin signaling tends to deregulate as inflammatory reactions increase, immunosurveillance against pathogens and premalignant cells decreases, and the composition of the peri- and extracellular environment changes [37]. Cells release inflammatory cytokines that promote a chronic inflammatory state, contributing to age-related diseases such as cardiovascular and neurodegenerative conditions. Senescent cells also release harmful molecules, known as SASPs, which affect nearby cells. SASPs can be beneficial and harmful to tissue homeostasis at the same time; therefore, they must be tightly controlled. This trend is one of the key characteristics of aging and contributes to many age-related diseases, such as cardiovascular diseases, neurodegenerative diseases, and type 2 diabetes [44]. Endocrine communication, though still important, becomes less effective due to the decreased release of hormones such as GH, testosterone, and estrogen, as well as the reduced sensitivity of target cells [185]. Non-classical intercellular communication increases due to the release of a larger number of extracellular vesicles and exosomes, which transmit molecules such as RNA and proteins and contribute to the spread of senescence and inflammation in tissues, particularly at the cerebral level. This type of communication is primarily regulated by epigenetic mechanisms [203,204]. Additionally, juxtacrine signaling, involving direct ligand–receptor interactions between adjacent cells, plays a role in modulating cellular responses during aging. For example, IL-1A, considered a key regulator of paracrine senescence, regulates juxtacrine senescence. The IL-1A downregulation prevents the upregulation of IL-6 and IL-8 during senescence [205]. In many cases, communication via vesicles becomes a vehicle to disseminate pro-inflammatory or degenerative signals. Communication through gap junctions decreases with age, compromising coordination between cells in tissues such as the heart and brain. These changes in intercellular communication are thought to be central to the aging process and its associated pathologies [206].

**Figure 15:**
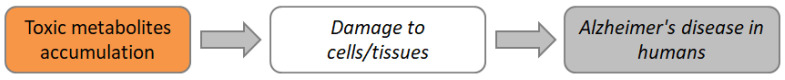
The buildup of toxic metabolites causes cellular and tissue damage, contributing to Alzheimer’s disease in humans.

- Accumulation of toxic metabolites (Figure 15).

As organisms age, some metabolic changes occur, leading to the accumulation of toxic metabolites, which are considered one of the hallmarks of aging. Cellular processes become less efficient at removing these compounds, leading to impaired cellular function and increased susceptibility to chronic disease. One of the key aspects of this process is metabolic dysregulation. Aging is associated with disruptions in core metabolic processes, resulting in the emergence of metabolites that reflect the body’s struggle to maintain metabolic homeostasis. These metabolites, such as advanced glycation end products (AGEs), tend to accumulate over time, contributing to cellular damage and dysfunction [207]. In addition, aging leads to an increase in oxidative stress, which is closely linked to the buildup of toxic metabolites. The accumulation of ROS can damage DNA, proteins, and lipids, thus contributing to a cycle of inflammation and further cellular damage [208]. In dogs, the accumulation of toxic metabolites has also been associated with age-related diseases, such as CKD [209,210]. Also, changes in the metabolism of amino acids and fatty acids are observed with aging, coinciding with higher oxidative stress markers. This oxidative imbalance may exacerbate the accumulation of harmful metabolic byproducts [211]. The aging process influences specific metabolic pathways, such as the urea cycle, and results in decreased levels of branched-chain amino acids. These changes lead to the buildup of toxic intermediates, further highlighting the connection between aging and the accumulation of damaging compounds [212]. The liver’s ability to process and neutralize these toxic substances declines, and toxic metabolites accumulate; thus, hepatotoxicity is a critical concern in dog aging [30,213].

These findings collectively highlight that the accumulation of toxic metabolites is increasingly recognized as a fundamental feature of aging, driven by metabolic and oxidative dysfunctions and reduced detoxification capacity. This buildup of harmful substances may accelerate cellular aging and contribute to the development of age-related diseases and functional decline as well.

**Figure 16:**
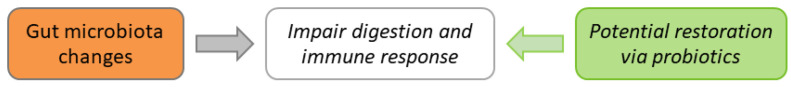
Aging disrupts gut microbiota, weakening immunity; probiotics may help restore balance and improve senior dog health.

- Dysbiosis (Figure 16).

Dysbiosis refers to an imbalance in the composition of the gut microbiota, which can affect host health. A healthy microbiota is essential for maintaining optimal immune and metabolic functions. Recent studies in humans have linked dysbiosis to various age-related conditions, including gastrointestinal disorders, autoimmune diseases, and metabolic disorders [214,215]. Among the predominant bacteria in the intestines of dogs, the number and prevalence of lactobacilli tend to decrease in older dogs. Research has demonstrated that a healthy microbiota can enhance systemic inflammation and support immune health, while dysbiosis has been associated with neurodegenerative diseases such as Parkinson’s disease [216,217]. In dogs as well, dysbiosis has been linked to gastrointestinal diseases and other age-related conditions. An altered microbiota in senior dogs may lead to digestive issues and affect immune response, similar to what has been observed in humans [218]. Recent studies suggest that probiotics may serve as potential interventions for restoring healthy microbiota and improving overall health in senior dogs [219,220]. Changes in gut microbiota are observed with aging, and since a healthy microbiota is essential in maintaining intestinal barrier integrity and modulating immune responses, dysbiosis can result in a weakened immune response and increased susceptibility to infections [221,222,223].

## 3. Conclusions

This review provides a comprehensive framework for assessing the health of senior animals and offers important clinical implications. Targeted interventions addressing specific hallmarks, such as chronic inflammation and mitochondrial dysfunction, can significantly improve the quality of life for senior dogs. The recognition of the interaction between environmental and genetic factors in aging remains pivotal. Modulating epigenetic mechanisms through interventions in the living environments of these animals may represent a promising strategy for enhancing the quality of physiological aging. While direct intervention in the genetics of adult animals is not feasible, epigenetic regulation offers a potential lever to promote long-term health and well-being. Furthermore, promoting education and awareness among pet owners and veterinary professionals about the hallmarks of aging in dogs is essential to optimize the quality of life for senior animals. By incorporating impaired water homeostasis as an additional hallmark, we underscore the essential role of water in maintaining physiological function in aging dogs. Water’s diverse roles—as a metabolic solvent, a transport medium for nutrients and waste, a regulator of body temperature, a structural component in cells, a mechanical protector for joints and tissues, and a mediator of electrical signals in neurons—become increasingly critical as dogs age. Maintaining proper hydration is thus a cornerstone for mitigating the impacts of aging, as dehydration can exacerbate issues such as cognitive dysfunction, tissue fragility, and metabolic inefficiency. This expanded understanding opens up promising avenues for innovative interventions aimed at preserving functional health and enhancing the quality of life in aging dogs. Prioritizing hydration as a fundamental target in managing the aging process may play a pivotal role in promoting their long-term vitality and well-being.

## Figures and Tables

**Table 1:** The table shows the key publications from the DAP highlighting the manuscript authors and year of publication, the objectives, main findings, and clinical implications. These studies contribute to a deeper understanding of canine aging and its translational relevance to human health and longevity.

Study	Objective	Key Findings	Clinical Implications
Creevy et al. (2022) [12]	Describe the objectives and design of the Dog Aging Project.	Introduction of a large-scale longitudinal study on aging in companion dogs.	Provides a foundation for understanding factors influencing aging in dogs with potential applications for human health.
Brayet al. (2022) [13]	Analyze the impact of diet on longevity and health in dogs.	Identification of correlations between specific dietary regimens and increased lifespan in dogs.	Proposes dietary guidelines to improve health and extend the lifespan of dogs.
Brayet al. (2023) [14]	Examine the effects of exercise on cognitive aging in dogs.	Regular exercise is associated with reduced age-related cognitive decline in older dogs.	Highlights the role of physical activity in maintaining cognitive function in aging dogs.
McCoy et al. (2023) [15]	Study the influence of environmental factors on canine aging.	Environmental factors like pollutant exposure and lifestyle significantly impact canine health and lifespan.	Stresses the importance of healthy environments in promoting aging healthily in dogs.
Morrill et al. (2022) [16]	Examine the relationship between canine breed genetics and behavioral traits.	Breed explains only a small portion of behavioral variation in individual dogs.	Challenges common stereotypes about breeds and suggests canine behavior is influenced by factors beyond breed genetics.
Fleming et al. (2019) [17]	Analyze causes of death in North American dogs from 1984 to 2004.	Identification of major causes of death linked to age, size, and breed in dogs.	Provides insights to improve healthcare management and preventive strategies in dogs.
Urfer et al. (2017) [18]	Evaluate the effects of short-term Rapamycin treatment in middle-aged dogs.	Rapamycin treatment was found to be safe and showed improvements in cardiac function.	Suggests potential interventions to extend lifespan and quality of life in dogs.

## Data Availability

This work has been funded by the European Union—NextGenerationEU under the Italian Ministry of University and Research (MUR) National Innovation Ecosystem grant ECS00000041—VITALITY project and the ‘Osteosarcoma project’ supported by University of Perugia—Research Fund 2022.
